# Supplementary material for: Splice-Junction-Based Mapping of Alternative Isoforms in the Human Proteome
Source: Cell Rep. Author manuscript; Available in PMC 2020 Jan 15. (PMC6961840; doi:10.1016/j.celrep.2019.11.026)
Supplement: 2 [file NIHMS1546469-supplement-2.zip › DF1/PXD010154/Testis/Testis_39_MLXIPL_AVTEGTLGKPGDSPAGLQVPRVAPSPDS.pdf]

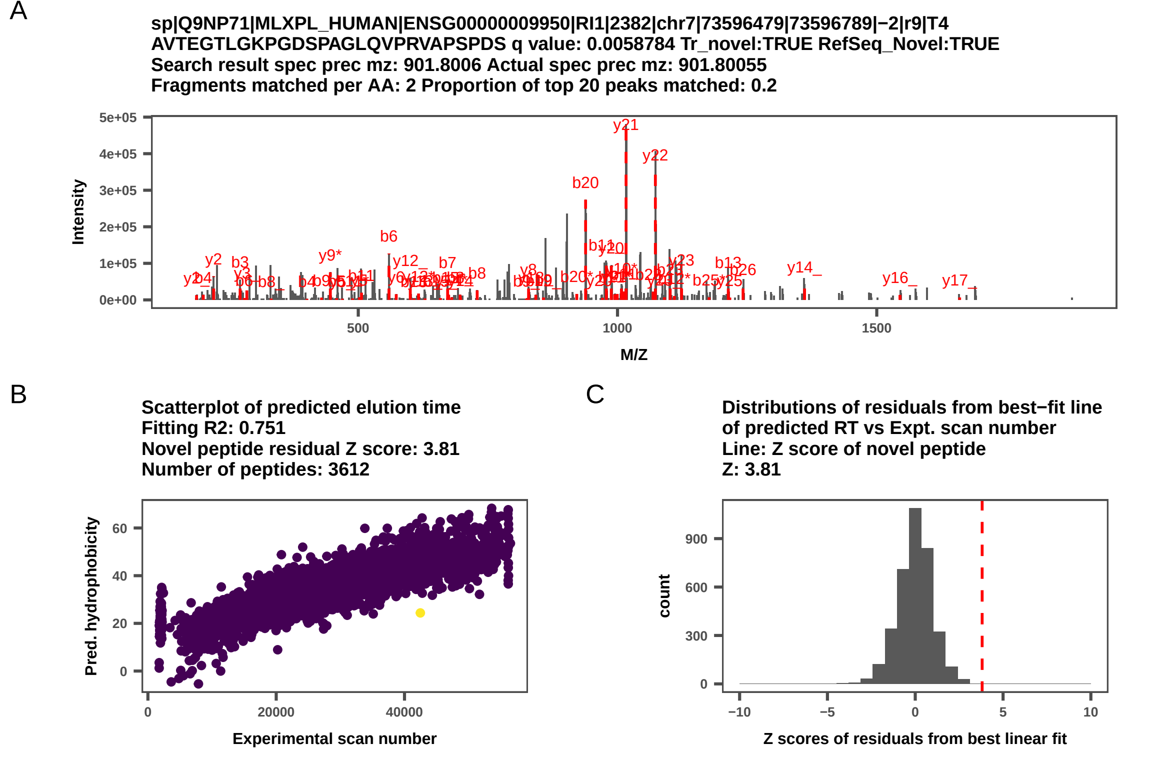
A
sp|Q9NP71|MLXPL_HUMAN|ENSG00000009950|RI1|2382|chr7|73596479|73596789|−2|r9|T4
AVTEGTLGKPGDSPAGLQVPRVAPSPDS q value: 0.0058784 Tr_novel:TRUE RefSeq_Novel:TRUE
Search result spec prec mz: 901.8006 Actual spec prec mz: 901.80055
Fragments matched per AA: 2 Proportion of top 20 peaks matched: 0.2
Intensity
M/Z
y21
y22
b20
b6
B
Scatterplot of predicted elution time
Fitting R2: 0.751
Novel peptide residual Z score: 3.81
Number of peptides: 3612
Pred. hydrophobicity
Experimental scan number
C
Distributions of residuals from best−fit line
of predicted RT vs Expt. scan number
Line: Z score of novel peptide
Z: 3.81
count
Z scores of residuals from best linear fit
